# What if HIV were unable to develop resistance against a new therapeutic agent?

**DOI:** 10.1186/1741-7015-11-249

**Published:** 2013-11-22

**Authors:** Mark A Wainberg, Thibault Mesplède, Francois Raffi

**Affiliations:** 1 Departments of Medicine and Microbiology, Jewish General Hospital, McGill University, Montreal, QC, Canada; 2 McGill University AIDS Centre, Montreal, QC, Canada; 3 Division of Infectious Diseases, Nantes University Hospital, Nantes, France

**Keywords:** Human immunodeficiency virus type 1, Integrase inhibitors, Antiretroviral therapy, Dolutegravir, HIV prevention strategies, Viral fitness, Drug resistance

## Abstract

**Background:**

The HIV integrase inhibitor, Dolutegravir (DTG), was recently approved by the Food and Drug Administration in the United States and is the only HIV drug that has not selected for resistance mutations in the clinic when used as part of first-line therapy. This has led to speculation that DTG might have a higher genetic barrier for the development of drug resistance than the other compounds that are used in therapy.

**Discussion:**

In this Opinion article, we speculate that this is due to greatly diminished replication capacity on the part of viruses that might become resistant to DTG when the drug is used in initial therapy and that DTG might be able to be used in HIV prevention and eradication strategies. We also note that no compensatory mutation that might restore viral replication fitness to HIV in the aftermath of the appearance of a single drug resistance mutation has yet to be observed.

**Summary:**

DTG is a valuable addition to the anti-HIV armamentarium of drugs and its long-term utility may potentially exceed its obvious use in treatment of HIV disease.

## Background

The current standard of care for treatment of HIV infection is the use of three antiretroviral (ARV) drugs in combination, with more and more simplified regimens becoming available. Since the introduction of triple ARV therapy in 1996, the rates of success of therapy, as indicated by suppression of plasma viremia to levels below a cut-off of 50 copies of viral RNA/ml, have increased to almost 90% [1]. This has happened for several major reasons. 1) The drugs used in therapy are now more potent and have longer half-lives than the compounds that were in use only 15 years ago. 2) Dosing regimens have become simplified, often because of the use of co-formulations, some of which only need to be taken once-daily, and this has greatly enhanced rates of adherence to ARV regimens. 3) Drug regimens have become far less toxic and more tolerable over time, and this has also promoted adherence as well as diminished the likelihood of development of HIV drug resistance against the components of ARV regimens [2,3].

The above notwithstanding, the use of ARVs in first line regimens has always been associated with some degree of treatment failure and drug resistance. Indeed, scientists have meticulously catalogued a wide array of drug resistance mutations that are located within each of the reverse transcriptase, protease and integrase of HIV-1 that are the targets of HIV therapy, and have documented how each of these mutations may lead to diminished likelihood of a favorable clinical response to each ARV, both in cell culture and in therapy [1]. The phase III clinical trials that led to the approval of each of the ARVs now used for therapy also provided valuable information on the types of viral mutations that were most likely to be identified in the event of treatment failure. This included studies on several of the most recent ARVs to have gained approval by regulatory agencies, most notably raltegravir (RAL) and elvitegravir (EVG) that are members of the integrase inhibitor family of drugs [4-9]. Now, however, a third member of this family, termed dolutegravir (DTG), has been studied in phase III clinical trials and has yielded the most robust results ever obtained in HIV registrational clinical trials [1,10,11]. First, approximately 88% of patients who received DTG together with two members of the nucleoside reverse-transcriptase inhibitor (NRTI) family of drugs in these studies attained suppression of viral load to <50 copies RNA/ml. Perhaps, more importantly, none of the individuals in the studies could be shown to possess a single drug resistance-related mutation. This is despite the fact that some patients in the trials, perhaps for reasons of non-adherence, did fail therapy and possessed detectable levels of viral load in plasma [9-11]. The other compounds employed were co-formulations of either lamivudine (3TC)/abacavir or emtricitabine (FTC)/tenofovir.

### The viral fitness hypothesis

**Figure 1:**
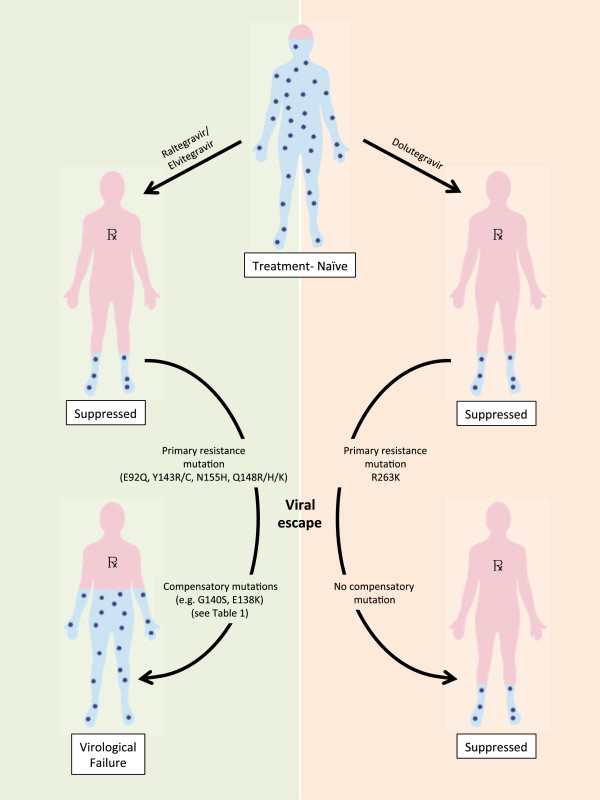
**Potential evolution of HIV-1 following therapy of previously treatment-naïve individuals with integrase inhibitors.** In rare cases, the emergence of resistance mutations in patients treated with raltegravir or elvitegravir can lead to virological failure (left). Virological failure with resistance mutations in treatment-naïve patients treated with dolutegravir has not been reported (right).

One hypothesis that has been advanced to explain these results is that viruses that become resistant to DTG may be relatively replication incapacitated and may be unlikely to efficiently grow or to be detected in patient samples [12]. Indeed, it has been shown that DTG can select a mutation at position R263K in the integrase gene in tissue culture and that this mutation diminishes both viral replication capacity as well as the enzymatic activity of the integrase enzyme [13]. This is in itself not unusual, as similar results had also been obtained with the two other approved integrase inhibitors RAL and EVG [4]. However, in the case of the latter two compounds, the presence of an initial mutation was often quickly followed by the appearance of a second substitution that had the dual effect of increasing the level of drug resistance, often to a level that might preclude any further clinical benefit from the drug, while simultaneously restoring viral replication capacity to normal levels (Figure 1, Table 1). In contrast, the secondary mutations that were selected by DTG only modestly increased overall levels of resistance against the drug but simultaneously impacted even more adversely on the ability of the virus to replicate. This was also reflected in a further diminution in the activity of the HIV integrase enzyme [12,13].

**Table 1:** Major resistance pathways for currently available INSTIs

**Raltegravir/Elvitegravir**	**Dolutegravir**
**Y143 pathway**	
Y143C	
Y143R	
T97A/Y143C	
T97A/Y143R	
L74M/T97A/Y143G	
L74M/T97A/E138A/Y143C	R263K
**N155H pathway**	
N155H	
L74M/N155H	
E92Q/N155H	
**E92Q pathway**	
E92Q	
T66I/E92Q	
E92Q/S153A	
E92Q/H51Y/L768V	
**Q148 pathway**	
Q148H	
Q148K	
Q148R	
E138K/Q148H	
E138K/Q148K	
E138K/Q148R	
G140S/Q148H	
G140S/Q148K	
G140S/Q148R	
E138A/G140S/Y143H/Q148H	

INSTIs, Integrase Strand Transfer Inhibitors.

The concept that secondary and/or tertiary drug resistance mutations may play a compensatory role in regard to replication is not unique to HIV. Indeed, similar findings have been documented for bacteria that are resistant to numerous antibiotics as well as for other viruses that display resistance against specific viral agents. In the case of HIV, compensatory mutations that simultaneously augment viral replication while increasing overall levels of drug resistance have been documented for members of each of the NRTI and non-nucleoside RT inhibitor (NNRTI) families of drugs as well as for protease inhibitors (PIs) and entry inhibitors [2]. Although treatment failure in the absence of resistance mutations in the viral protease gene has been described, this is widely attributed to the fact that cleavage site mutations in the Gag and Gag-pol substrates of the protease enzyme are known to occur, and such mutations, although not well characterized, are known to confer resistance to PIs.

The fact that no resistance mutation has yet been identified for DTG in treatment-naïve patients represents a unique observation. Moreover, this finding is bolstered by the results of tissue culture selection experiments with DTG that have only yielded two distinct mutations that are associated with diminished viral replicative capacity but never a third compensatory mutation, despite efforts that have persisted over almost three years [12]. Therefore, an added benefit of using DTG in first-line therapy may be that viruses that do contain R263K will not be transmitted or that this mutation will revert if viral transmission does take place, due to low replication fitness. Such reversion has been reported for HIV variants that contain the M184V mutation that is associated with resistance to 3TC and FTC and that also impairs HIV fitness. It may turn out, as an example, that the use of DTG in Treatment as Prevention (TasP) protocols, whereby reductions in viral load on a population level can be expected to result in diminished rates of HIV transmission, will obviate concerns about the development of resistance to the drugs used in this strategy.

This raises several issues. First, what if it turns out that viruses that are resistant to DTG cannot be compensated by additional mutations within Integrase and that such viruses are at a severe replication disadvantage in comparison with wild-type viruses. This result would take on even greater significance should it turn out that DTG can retain clinically significant antiviral activity, despite the presence of one or two mutations associated with drug resistance. In fact, such a scenario is suggested by the fact that the level of resistance conferred against DTG by the combination of two mutations within Integrase is relatively slight, that is, <10-fold. Furthermore, biochemical results have shown that the ability of DTG to bind to the Integrase enzyme and remain associated with it is very long, that is, >36 hours, and that the R263K mutation only diminishes this level of binding by about 50% [14]. While this may seem substantial, the reality is that this is still longer than the binding affinity half-life of RAL for the wild-type integrase enzyme. Could these findings in fact suggest that the development of resistance against DTG might at the end turn out to provide a unique virologic and clinical benefit?

One way to test this notion might be to conduct a study in which DTG is employed as monotherapy in treatment inexperienced subjects. Should it turn out that the results obtained are similar to those observed in the phase III clinical trials, a partial validation of the hypothesis to explain the absence of resistance in the phase III trials will have been provided. Of course, such a monotherapy study would need to be accompanied by intense virologic monitoring for resistance mutations, which should include the use of ultrasensitive sequencing methods for identification of DTG resistance mutations in both the RNA of patient plasma samples as well as in the DNA of patient peripheral blood mononuclear cells.

In one sense, some clinical validation of the significance of the R263K mutation has already been obtained. Notably, the SAILING-clinical trial compared the use of RAL against DTG in treatment-experienced patients who had undergone previous failures of their therapeutic regimens but who had never before been treated with an integrase inhibitor [15]. Many of these patients possessed drug resistance mutations that might have compromised the anti-viral activity of multiple ARVs in the regimens that they received in the SAILING study, but not of the integrase inhibitors. The results of the trial showed that DTG was superior to RAL at suppression of viral load in this population. Moreover, the only drug resistance mutation to have appeared in only very few patients in the DTG arm of the study was R263K. Although this cautions that the development of resistance to DTG in drug-naïve patients may be possible, it should be noted that the patients who received DTG and who possessed the R263K mutation continued to do very well from a clinical perspective over the 48-week period of the trial. Failure on the RAL arm of the study led to a broad array of mutations in Integrase that are associated with resistance to the latter drug.

Based on these observations, a strong case can be made that DTG can be considered as a drug of choice for patients entering therapy for the first time. Although the development of R263K and a subsequent mutation may not confer any deleterious effect in regard to patient management, it is clear that the prior development of mutations associated with resistance against RAL or EVG may compromise the clinical performance of DTG. Each of the Viking I, II and III studies has now shown that DTG can be successfully used to salvage significant numbers of patients who first were treated with RAL or EVG and who failed those regimens [16]. However, a successful clinical outcome was not accomplished in many cases, and there seems little doubt that many patients who first fail RAL- or EVG-based regimens may not be able to remain durably suppressed virologically when treated with DTG as part of a second-line regimen. The argument that integrase inhibitors can or should always be used sequentially, beginning with a different drug, such as RAL or EVG, and then switching to DTG, may not be sustainable.

### Future directions

A more intriguing question, however, is what will happen if patients do as well on DTG monotherapy as on triple therapy, despite the presence of the R263K mutation. Would clinicians then be willing to entertain the notion of withholding DTG from therapy at a certain point as part of a structured treatment interruption? In this scenario, it is conceivable that the impaired viruses containing DTG resistance mutations would not be able to grow out. What would then become of the wild-type viruses that had become archived after infecting the patient in the first place? Presumably, a high proportion of such viruses would begin to replicate following activation of latent reservoirs in the same manner as has been observed following treatment interruption in other trials. However, re-initiation of DTG monotherapy might then convert these wild-type viruses into DTG-resistant attenuated forms. Is it conceivable that a number of cycles of DTG treatment interruption followed by re-initiation of DTG monotherapy could convert all the HIV in the body to a replication impaired form? Could such an approach lead to a functional cure of HIV disease if all residual viruses were significantly impaired in viral replication and if further compensatory mutations were unable to develop?

To be sure, these are the types of concepts that should ideally first be studied in animal models such as rhesus macaques that are infected by simian immunodeficiency virus (SIV) or humanized mice that are infected by HIV. However, some clinicians have experimented with monotherapy in the past and are likely to do so again. Moreover, there is a probability that this will happen with DTG after it is approved by regulatory agencies, when there are fewer limitations on the conduct of small-scale clinical trials of this type. There is a likelihood that such studies will be ethically justifiable, if a case can be made for benefits that exceed those of the suppression of viral load.

## Discussion

One last issue relates to possible implications for the companies that plan to sell DTG if this drug is successfully used in either HIV cure or prevention strategies. For one thing, the cost of DTG has already been established in virtually all countries at a level that is based on the price of other currently approved ARVs and the expectation that patients will need to take DTG on a chronic basis over many years. Although noone would want the cost of DTG to be increased, the reality is that the cost of treatment with potentially curative drugs for hepatitis C virus is likely to be at least five times that of DTG. In addition, the pressures to make a potentially unprecedented treatment for HIV available as expeditiously as possible for people in developing countries will be difficult to withstand. Of course, as stated above, these scenarios may only apply to patients who have not previously been treated with any integrase inhibitor and for whom the viral fitness hypothesis following use of DTG makes good sense.

## Summary

Dolutegravir is a welcome addition to the anti-HIV armamentarium of drugs and has shown unprecedented benefit to patients who have taken it as part of a first-line therapeutic regimen. Until now, no resistance mutation has developed either against DTG or any of the drugs used together with it in first-line therapy, and supportive tissue culture data have shown that the development of an initial HIV resistance mutation against DTG may result in a virus with greatly diminished replicative fitness. It is possible that DTG will lend itself to use in a variety of HIV prevention strategies, such as Treatment as Prevention, and that it might possibly also be used in efforts designed to accomplish a cure for HIV infection.

## Abbreviations

3TC: Lamivudine; ARV: Antiretroviral; DTG: Dolutegravir; EVG: Elvitegravir; FTC: Emtricitabine; HIV-1: Human immunodeficiency virus type 1; NRTI: Nucleoside reverse-transcriptase inhibitor; PIs: Protease inhibitors; RAL: Raltegravir.

## Competing interests

Mark A. Wainberg and Francois Raffi have received research grant support from each of the following companies: ViiV Healthcare, Merck Inc., Bristol-Myers Squibb, Gilead, Abbvie and Janssen Pharmaceuticals. Thibault Mesplède has no competing interests.

## Authors’ contributions

Each of the authors contributed to the writing of this manuscript and all have approved its final version.

## Authors’ information

Mark A. Wainberg is Professor of Medicine, McGill University, Montreal, QC, Canada; Thibault Mesplède is a Research Associate, McGill University AIDS Centre, Montreal, QC, Canada; and Francois Raffi is Professor and Director of the Division of Infectious Diseases, Nantes University Hospital, Nantes, France.

## Note

This article is part of the cross journal collection HIV 30 years on. Other articles in this series can be found at http://www.biomedcentral.com/series/HIV_30.
